# AFM as an analysis tool for high-capacity sulfur cathodes for Li–S batteries

**DOI:** 10.3762/bjnano.4.68

**Published:** 2013-10-04

**Authors:** Renate Hiesgen, Seniz Sörgel, Rémi Costa, Linus Carlé, Ines Galm, Natalia Cañas, Brigitta Pascucci, K Andreas Friedrich

**Affiliations:** 1 Faculty of Basic Science, University of Applied Sciences Esslingen, Esslingen, Germany,; 2 Institute of Technical Thermodynamics, Electrochemical Energy Technology, German Aerospace Center, Stuttgart, Germany

**Keywords:** conductive AFM, high capacity, lithium-sulfur batteries, material-sensitive AFM, sulfur cathode

## Abstract

In this work, material-sensitive atomic force microscopy (AFM) techniques were used to analyse the cathodes of lithium–sulfur batteries. A comparison of their nanoscale electrical, electrochemical, and morphological properties was performed with samples prepared by either suspension-spraying or doctor-blade coating with different binders. Morphological studies of the cathodes before and after the electrochemical tests were performed by using AFM and scanning electron microscopy (SEM). The cathodes that contained polyvinylidene fluoride (PVDF) and were prepared by spray-coating exhibited a superior stability of the morphology and the electric network associated with the capacity and cycling stability of these batteries. A reduction of the conductive area determined by conductive AFM was found to correlate to the battery capacity loss for all cathodes. X-ray diffraction (XRD) measurements of Li_2_S exposed to ambient air showed that insulating Li_2_S hydrolyses to insulating LiOH. This validates the significance of electrical ex-situ AFM analysis after cycling. Conductive tapping mode AFM indicated the existence of large carbon-coated sulfur particles. Based on the analytical findings, the first results of an optimized cathode showed a much improved discharge capacity of 800 mA·g(sulfur)^−1^ after 43 cycles.

## Introduction

Lithium rechargeable batteries with high capacity are a key technology for the widespread implementation of battery-powered cars. The specific energy of existing lithium batteries needs further improvement to enable acceptable driving ranges of electric vehicles. Moreover, this is also important for portable applications. Besides efficiency and energy density, numerous other requirements have to be fulfilled, which include industrial scalability and the capability for mass-production. In the last few decades, lithium–sulfur batteries have attracted increasing attention due to their high theoretical energy density (2500 Wh·kg^−1^) and theoretical capacity (1672 mA·g^−1^), which are based on the electrochemical reaction 16 Li + S_8_ 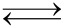 8 Li_2_S [1–2]. In addition, these batteries have the advantage of having sulfur-based cathodes, which are cheap, abundant, and environmentally friendly. However, these batteries suffer from much lower realised capacities and lifecycles, which is mainly due to:

(1) the low electrical conductivity of sulfur (5 × 10^−30^ S·cm^−1^ at 25 °C), which leads to a poor electrochemical accessibility and a low utilisation of sulfur,

(2) the electrochemical irreversibility due to the loss of sulfur active-material and parasitic reactions of dissolved polysulfides at the Li electrode and

(3) the morphological and volumetric changes of the cathode material upon cycling [3–4].

The redox reaction of the sulfur cathode can only occur when the sulfur is in contact with the carbon because of the insulating nature of sulfur. In this regard, an ideal cathode would be composed of a continuous, electronically conductive carbon network coated with a monolayer of sulfur. The contact between the carbon–sulfur composite and the current collector is also a very important parameter for the performance of the Li–S battery, which depends mainly on the type of binder that is used [5–7].

Related to the morphology and volume changes of the cathodes, it was found that the sulfur cathodes expand while discharging and shrink while charging. The thickness change of the electrode was measured to be approximately 22% [8]. Capacity fading due to structural and volume changes was reported in several publications [4,9–13]. Therefore, to achieve a high performance Li–S battery, it is necessary to restrict the changes in the morphology and volume of the cathode. Recent advances with graphene–sulfur composite materials demonstrated reasonably high and stable specific capacities of up to 600 mA·g(sulfur)^−1^ over more than 100 cycles [13–14]. One way to suppress the polysulfide shuttle mechanism and to enhance the sulfur retention is to coat the electrodes. This can be performed by physical vapour deposition of a nickel layer or by coating with Nafion [15–16]. To obtain a superior capacity and reversible cycle performance, the production of thin and porous sulfur cathodes or the use of foam-like structures as current collectors have been shown to be advantageous [9,17–18]. Recent studies have shown that the use of highly ordered mesoporous carbon with a bimodal pore structure with a high specific area and a large pore volume is beneficial. It traps a part of the polysulfides for a certain time before release, thereby reduces the electrochemical irreversibility and can lead to a very high and stable capacity of approximately 1000 mA·g(sulfur)^−1^ [19–20]. Another approach is based on vertically aligned carbon nanotubes (CNTs) grown on a nickel foil without any binder. To date, these binder-free CNT cathodes contain the highest published total ratio of sulfur (90%) in an electrode [21]. The advantage of a stable three-dimensional conductive network achieved by the introduction of carbon nanofibres has also been demonstrated [22]. Besides these more sophisticated approaches the introduction of a porous carbon/polytetrafluorethylen (PTFE) containing material, which is used as a gas diffusion layer in fuel cells (GDL), positioned in front of the cathode has led to capacities, in dependence on the discharge rate, of 1000–1200 mA·g(sulfur)^−1^ [23].

In this work, the aim is to investigate the electrical and morphological stability of lithium–sulfur cathodes manufactured by suspension spraying or doctor blading in order to improve the performance of these electrodes. Results from recent XRD studies were used that clarified a complete chemical reaction of the non-conductive Li_2_S layer to an also insulating LiOH layer. This layer was stable in air and enabled an AFM study of the conductive area [24]. Polyvinylidene fluoride (PVDF) and carboxymethyl cellulose (CMC) binders were used to test the influence of the binders on the battery performance. The suspension-spraying technique is advantageous because it can be used in mass production processes. In a first step the focus was on the preparation of a porous, homogeneous, thin, and agglomeration-free cathode, which exhibited reduced structural changes during the discharge–charge cycles. Morphological changes and the stability of the electronic conductivity of the sulfur cathodes upon cycling were detected by means of SEM, material-sensitive AFM and conductive tapping mode AFM. We aim to demonstrate that a direct correlation exists between the cycling stability and the properties on the nanometre scale, and that AFM analysis can disclose the morphology of the carbon–sulfur interface. Based on the analytical results of the nanoscale analysis an optimized preparation technique was introduced which lead to an enhanced battery performance.

## Results and Discussion

### Li–S batteries with different preparation and composition

**Figure 1:**
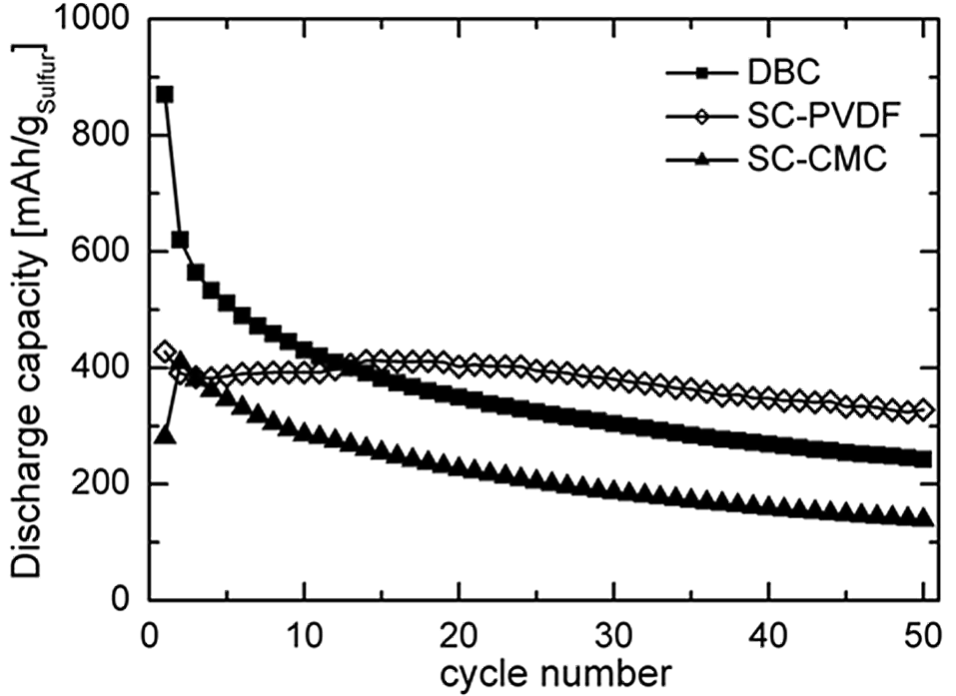
Comparison of the cycling performances of non-optimised Li–S batteries prepared with different binders. The batteries were tested in the range of 2.8–1.5 V vs Li/Li^+^ at a current density of 533 mA·g^−1^ sulfur.

Figure 1 shows a comparison of the discharge capacity of the first batteries containing differently prepared cathodes over 50 cycles.

Although the battery, whose cathode was coated by hand (doctor-blade coated, referred to as DBC-PVDF), has a high initial discharge capacity (872 mAh·g^−1^), the capacity decreases significantly upon cycling. After 50 cycles, the remaining capacity is only 242 mAh·g^−1^. This behaviour is quite typical for the Li–S cathodes [3,12,25–26].

In contrast to the DBC-PVDF sample, battery cathodes with the same composition but prepared by a home-made suspension spraying device (suspension coated, referred to as SC-PVDF) show a substantial reversible capacity of 330 mAh·g^−1^ after 50 cycles (see Figure 1). The degradation of the capacity after 100 cycles is only 25%, which demonstrates a good electrochemical reversibility. The better performance of the sprayed cathode can be attributed to the porous and homogeneous carbon–sulfur network-structure, which is mostly stable upon cycling, as proofed by SEM and AFM measurements (see below).

The sprayed cathodes prepared with carboxymethyl cellulose (CMC) binders (SC-CMC) show discharge capacities of 136 mAh·g^−1^ after 50 cycles, which is inferior to other values (Figure 1). One of the reasons for the low initial discharge capacity compared to the theoretical capacity (1672 mAh·g^−1^) could be the bad contact between sulfur and carbon black, so that not all of the sulfur in the cathode is reduced (low sulfur utilisation). Another reason could be the non-ideal penetration of the electrolyte into the cathode. If the cathode is not ideally porous, or if the cathode is too thick, and if the amount of electrolyte is insufficient to fill the pore volume, sulfur is only partially reduced. It is therefore of interest to optimise the amount of electrolyte as a function of the free volume available within the porous cathode. Concerning the capacity fading observed on the tested cathodes, several hypotheses can be proposed:

(1) During battery discharge, elemental sulfur (S_8_) is first broken down to form a chain-like polysulfide anion (S_8_^2−^), which combines with Li^+^ ions to yield high-order lithium polysulfides (Li_2_S*_x_*, *x* ≥ 4). These high-order polysulfides are soluble in the organic electrolytes and therefore the viscosity of the electrolyte increases [27]. In this case, Li^+^ ion diffusion and penetration into the inner parts of the cathode decrease. Therefore, most of the reduction reactions take place on the surface of the cathode.

(2) Some of the reduction products, Li_2_S_2_ and Li_2_S, which are low-order polysulfides, are insoluble in the electrolyte and stay on the surface of the cathode to form a dense film [9,13,28–30]. During these processes the carbon–sulfur network is subject to modification. Therefore, the electronic percolation is partially reduced, which results in the formation of electrochemically inactive areas. For this reason, low-order polysulfides cannot be oxidised back to high-order polysulfides within these areas. Additionally, these low-order polysulfides block the pores of the cathode upon cycling and the electrolyte cannot penetrate properly into the cathode structure. Therefore, Li^+^ ion diffusion is reduced and further electrochemical reactions are restricted. All of these phenomena result in degradation of the capacity.

(3) Some of the high-order polysulfides migrate through to the anode side due to the shuttle mechanism and react with Li^+^ ions on the surface of the anode [31–32]. This time, low-order polysulfides form and settle down on the surface of the lithium anode. They cannot be oxidised back and therefore block the active sites of the anode surface [31].

**Figure 2:**
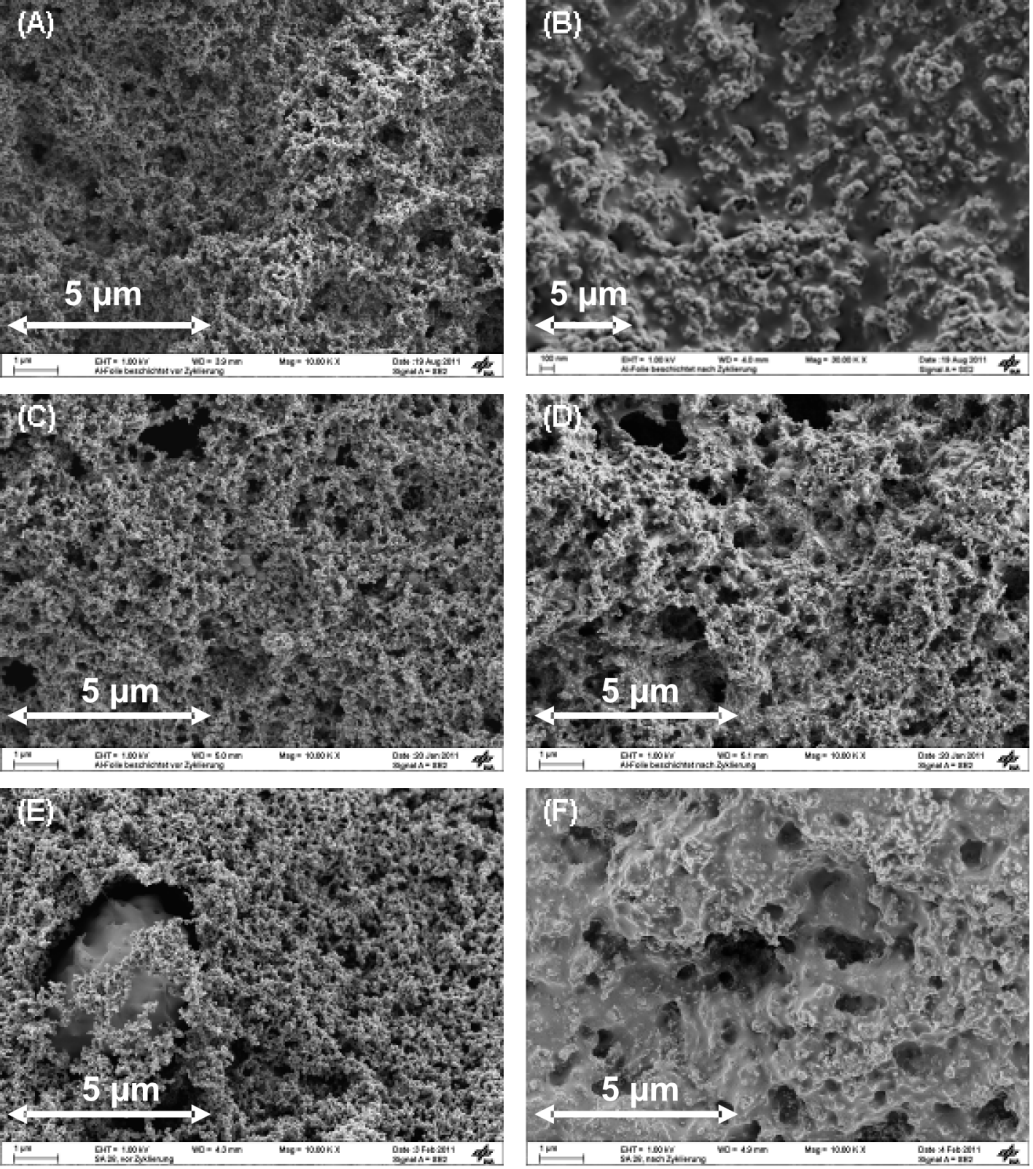
SEM images of (a) DBC-PVDF before cycling, (b) DBC-PVDF after the 50th discharge, (c) SC-PVDF before cycling, (d) SC-PVDF after the 50th discharge, (e) SC-CMC before cycling, (f) SC-CMC after the 50th discharge.

As shown in Figure 2c and Figure 2d, the morphological changes upon cycling of the SC-PVDF cathode are much less than those of the DBC-PVDF (Figure 2a and Figure 2b) or SC-CMC cathodes (Figure 2e and Figure 2f). The porous structure of the SC-PVDF cathode is preserved after 50 discharge/charge cycles. In this way, further electrochemical reactions are possible.

Although the SC-CMC samples were prepared by spraying, it was not possible to obtain a reversible capacity, which is most likely due to the formation of a crust-like layer on the cathode surface upon cycling (Figure 2f). As one can see in Figure 2e, the application of a CMC binder in a sulfur cathode caused the formation of loose particles and also led to a bad contact between carbon black and the sulfur particles. Therefore, the carbon–sulfur network structure was not stable upon cycling and changes of morphology and volume were observed. A proper binder should have a high adhesion between the electrode materials and the current collector and should form a good network between the active material and the conductive carbon. In this way, the electron transport as well as the diffusion of the lithium ions is facilitated [33].

### X-Ray diffraction

**Figure 3:**
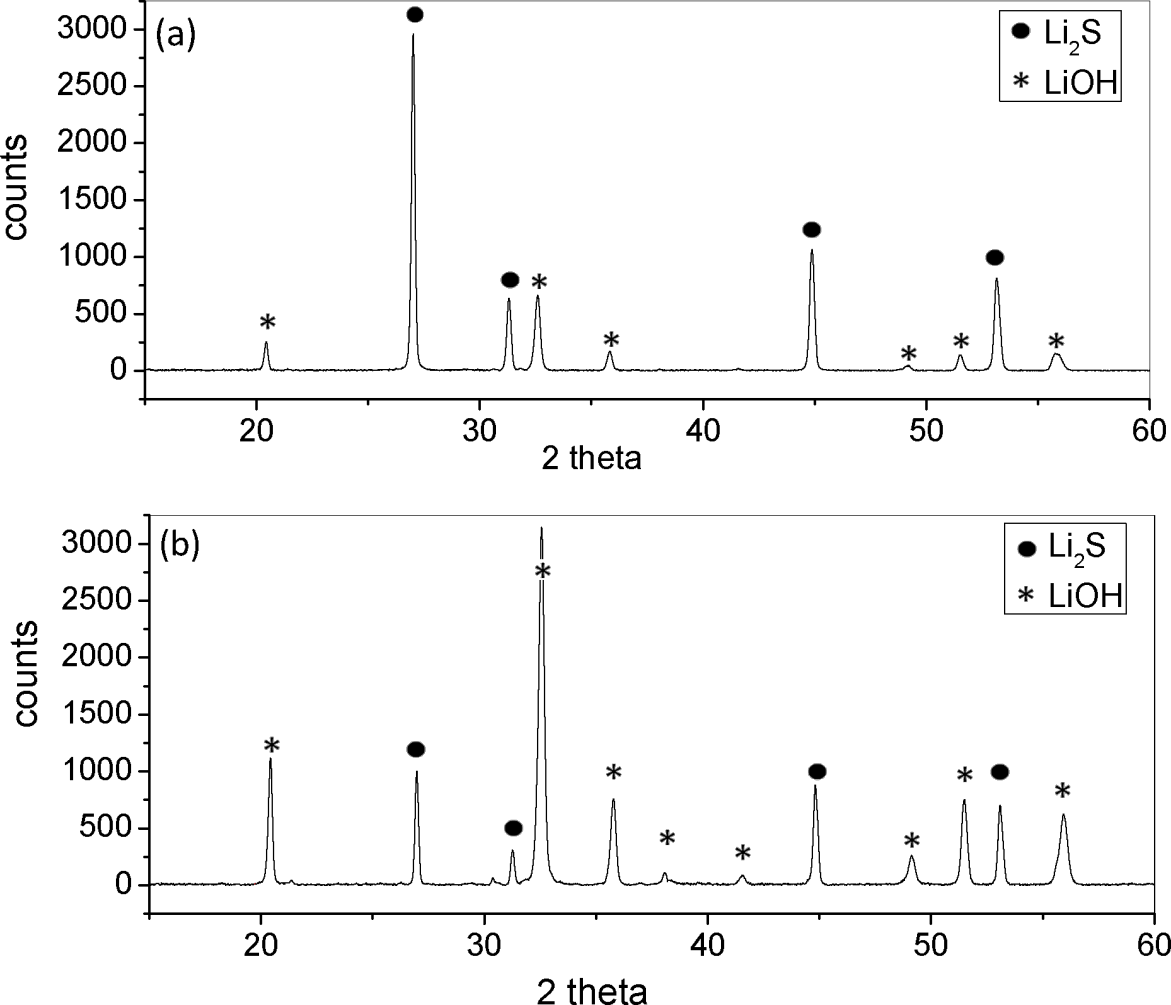
X-ray pattern of the Li_2_S sample before (a) and after (b) approximately 25 min in contact with air.

Lithium containing components like the cathodes after cycling in a battery are sensitive to ambient air. Water as well as nitrogen and oxygen reacts with the lithium and change surface and bulk composition. Therefore AFM analysis of battery materials, which contain lithium, has been performed in situ in a glove-box [11]. Upon cycling a reduction of the conductive area was observed that was attributed to the formation of a non-conductive Li_2_S layer. The stability of Li_2_S in air was analysed by means of XRD. Li_2_S powder was exposed to air, and the subsequent reaction was measured in a time-dependent sequence. Figure 3 shows the X-ray patterns of the Li_2_S sample before (a) and after (b) approximately 25 min of contact with air. In air, lithium sulfide easily hydrolyses and reacts to form hydrogen sulfide and lithium hydroxide (Li_2_S + 2 H_2_O → 2 LiOH + H_2_S). It can be observed that the integrated intensity of the peaks of Li_2_S decreased significantly, while the peak intensity of LiOH increased. Instead of the insulating Li_2_S layer an also insulating LiOH layer was formed, which was stable in air. Measurements during exposure to air were also made by using AFM imaging. No significant differences in stiffness and in conductivity were detected. However, changes may have already happened during the transfer procedure. Therefore, an ex-situ analysis by using AFM allowed for retrieval of significant results on the percentage of the remaining conductive area after cycling. Details of these measurements can be found in [24].

### Atomic force microscopy

#### AFM of basic components

**Figure 4:**
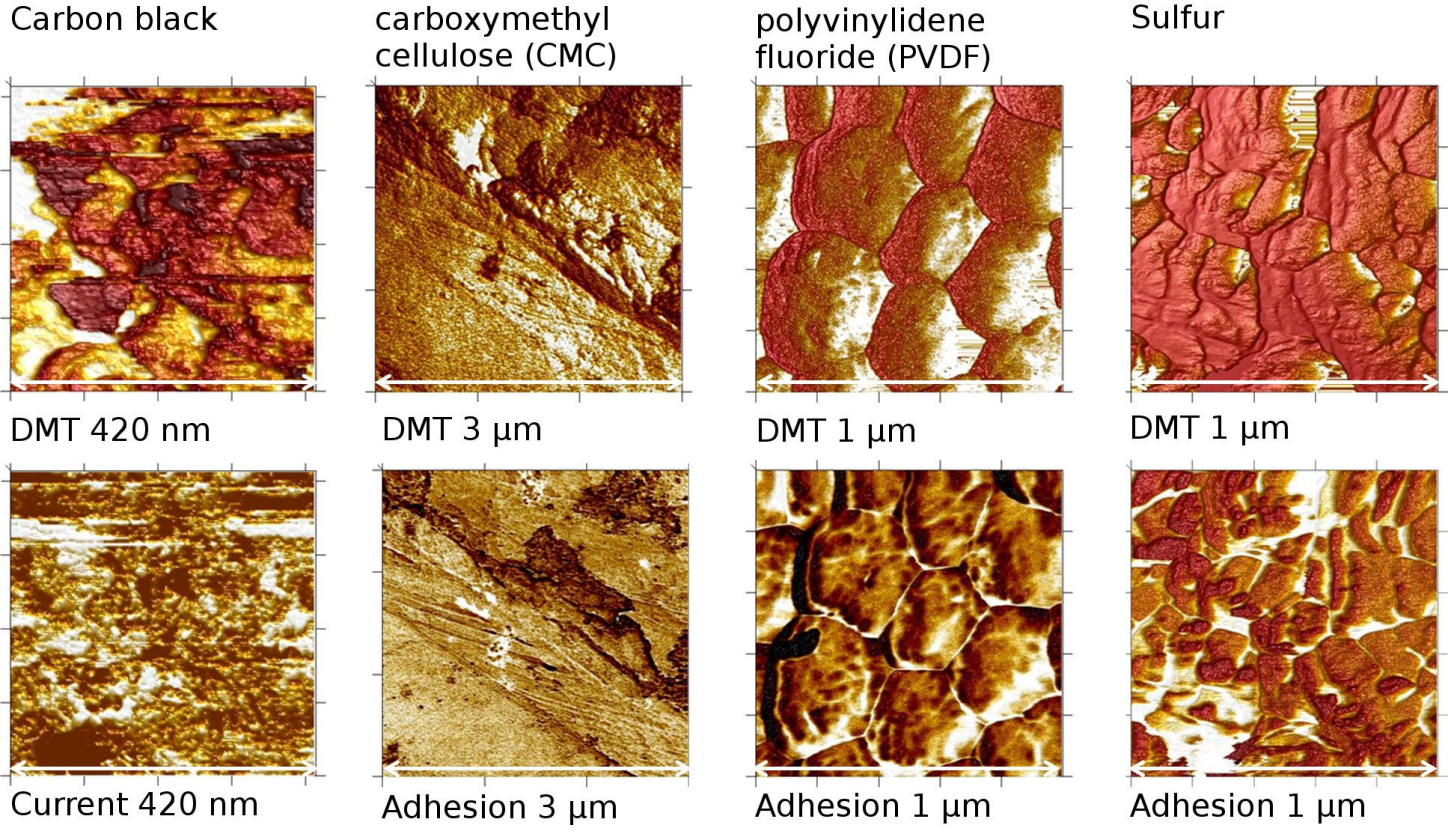
Mapping of DMT modulus and current/adhesion of basic materials used for cathode preparation from AFM, brighter colours indicate higher values. The size of the scale bar is given below each image.

In order to calibrate the AFM for the material-sensitive analysis, an evaluation of the properties of the basic materials, which were used for the cathode preparation, was performed. Therefore, pellets were prepared from powder at a pressure of 10 kN and imaged separately by AFM (Figure 4).

**Figure 5:**
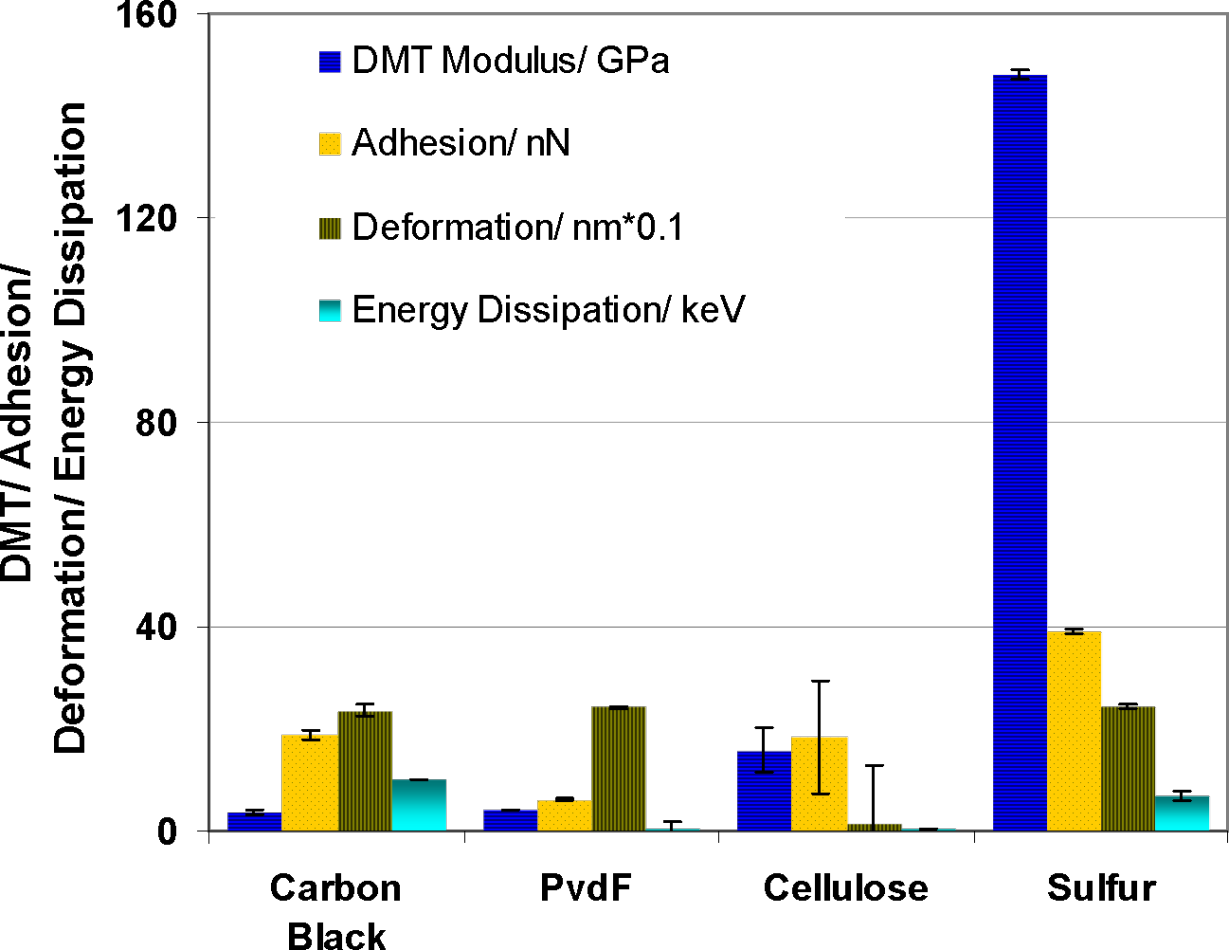
Statistical evaluation of mechanical properties of the basic materials used for preparing the cathodes.

As shown in Figure 5, the DMT modulus (stiffness) differs significantly. The highest values were measured for sulfur and a very small stiffness was measured for carbon, which was the only conductive sample in this series. In the other properties, sulfur differed slightly from carbon black and binder with a smaller energy dissipation and a slightly increased adhesion. It is noted, that the measured DMT modulus has values out of the recommended range for this type of tip. Therefore, the accuracy of those values is not very high. However, the large differences allowed a good differentiation from carbon. No significant differences were found for the deformation values with the exception of cellulose as a binder material. The adhesion force was smallest for the fluorine containing PVDF binder. For the identification of sulfur the stiffness values were used. During the stiffness measurements the tip puts a pressure on the surface and a subsurface volume is involved. Thereby, even if coated with a thin layer of other material, sulfur can be identified with high certainty. The identification of carbon is performed by measuring its conductivity.

**Figure 6:**
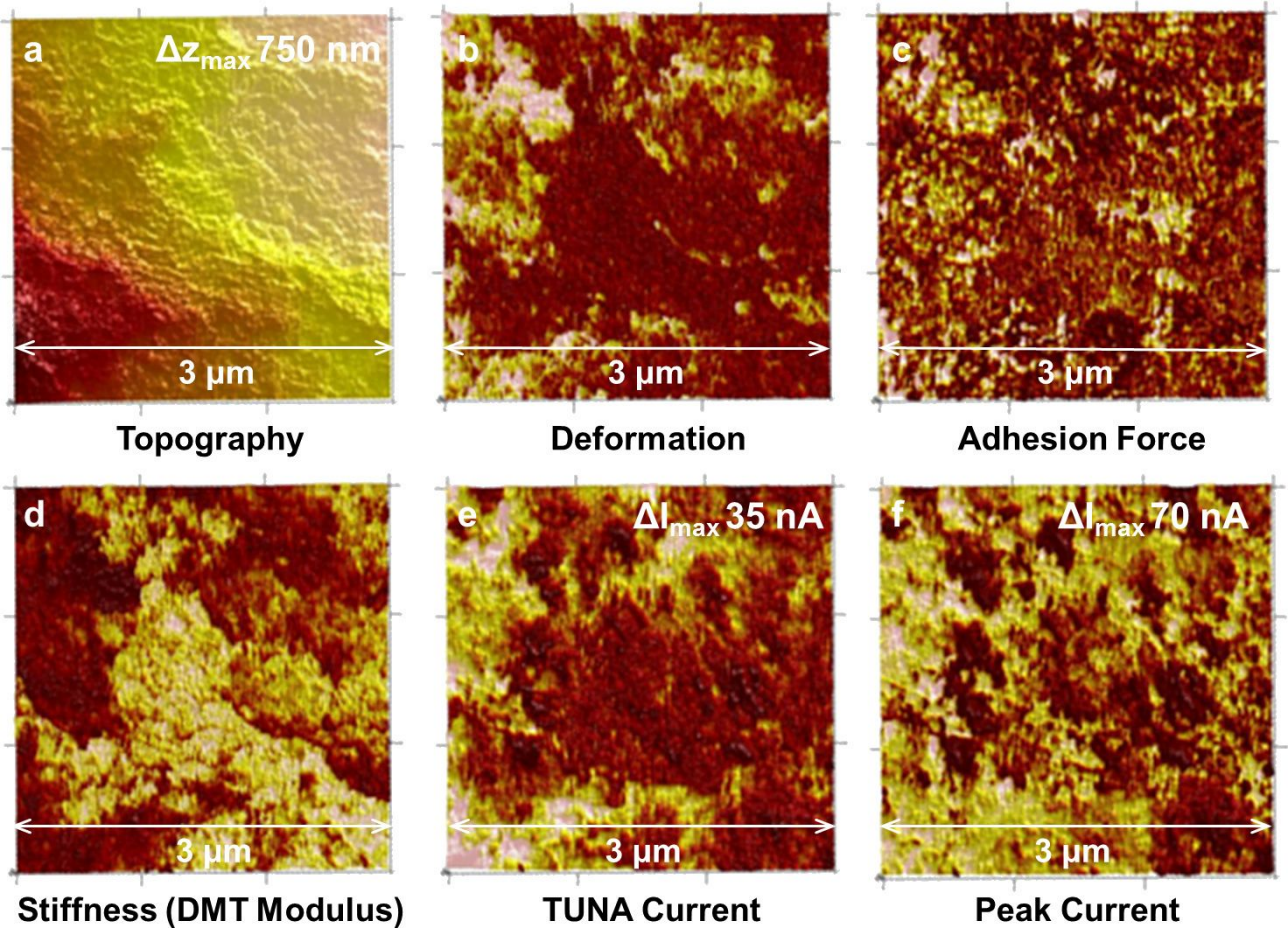
AFM images of an SC-PVDF sample before cycling. a) topography, b) deformation, c) adhesion, d) DMT modulus (stiffness), e) PeakForce-TUNA^™^ current, f) PeakForce QNM^™^ current (brighter colours are higher values).

In Figure 6 AFM images of the SC-PVDF sample are shown. All AFM images measure 3 μm × 3 μm. The topography is displayed together with the simultaneously measured mapping of deformation, adhesion force, DMT modulus (stiffness), TUNA™ current, and peak current. The different properties of image areas allowed for a distinction of different surface materials, which is not possible with the topography image only.

For all other samples these properties were measured and used for the analysis. However, for simplicity, only the topography images, together with stiffness and current images, recorded by conductive PeakForce-QNM™ (see section Experimental), are displayed. The surface is rough, consistent with the SEM images of Figure 2, with height differences of 750 nm on a 3 µm scale. A large part of the surface exhibits a comparably hard/elastic material with high DMT modulus (brighter colours indicate areas with higher stiffness). These regions concurrently exhibit a lower deformation. The adhesion force is higher at those parts of the surface where the stiffness values are smaller. In the centre of the TUNA current image in Figure 6, the current density has lower intensities, whereas the corresponding stiffness is especially high. Deformation values, on the other hand, are relatively low. Due to the insulating nature of sulfur, it can be expected that areas of high sulfur content exhibit low current density; however, they should have a high stiffness compared to carbon-rich areas (see Figure 5). It can therefore be concluded that a heterogeneous chemical distribution is present in this area, with a sulfur-rich region in the centre. No indication of this heterogeneity is visible in the topographical images. As observed in the recording of the TUNA™ current (where the average steady state current is displayed, Figure 6e), little or no steady current flow was measured at the parts of the surface with high stiffness (DMT). In contrast, a peak current (Figure 6f) was present in this area. The peak current signal gives the current flow at maximal pressure of the AFM tip (Δ*t* ≈ 0.001 s). In this time domain, transient (capacitive) currents can be detected and were present in most parts of the surface with high stiffness. These transient currents indicated surface regions where fast charging processes occurred even before contact of that sample to lithium species. Therefore, the transients were not associated with an ionic charging process. An electronic charge transfer is present and a charging of carbon agglomerates on insulating sulfur particles (indicated by the high stiffness of this region) with the tip is assumed. The size of the underlying sulfur particles retrieved from the high stiffness region is about 0.5 µm and presumably they are too large for a high utilization of sulfur. In the peak current image, several black spots indicate regions where no current was measured and concurrently displayed highest stiffness. By comparing the size of the carbon particles with the particles in the range of a few ten nanometers as reported by [34], the resulting coverage of large sulfur particles by the much smaller carbon is expected.

**Figure 7:**
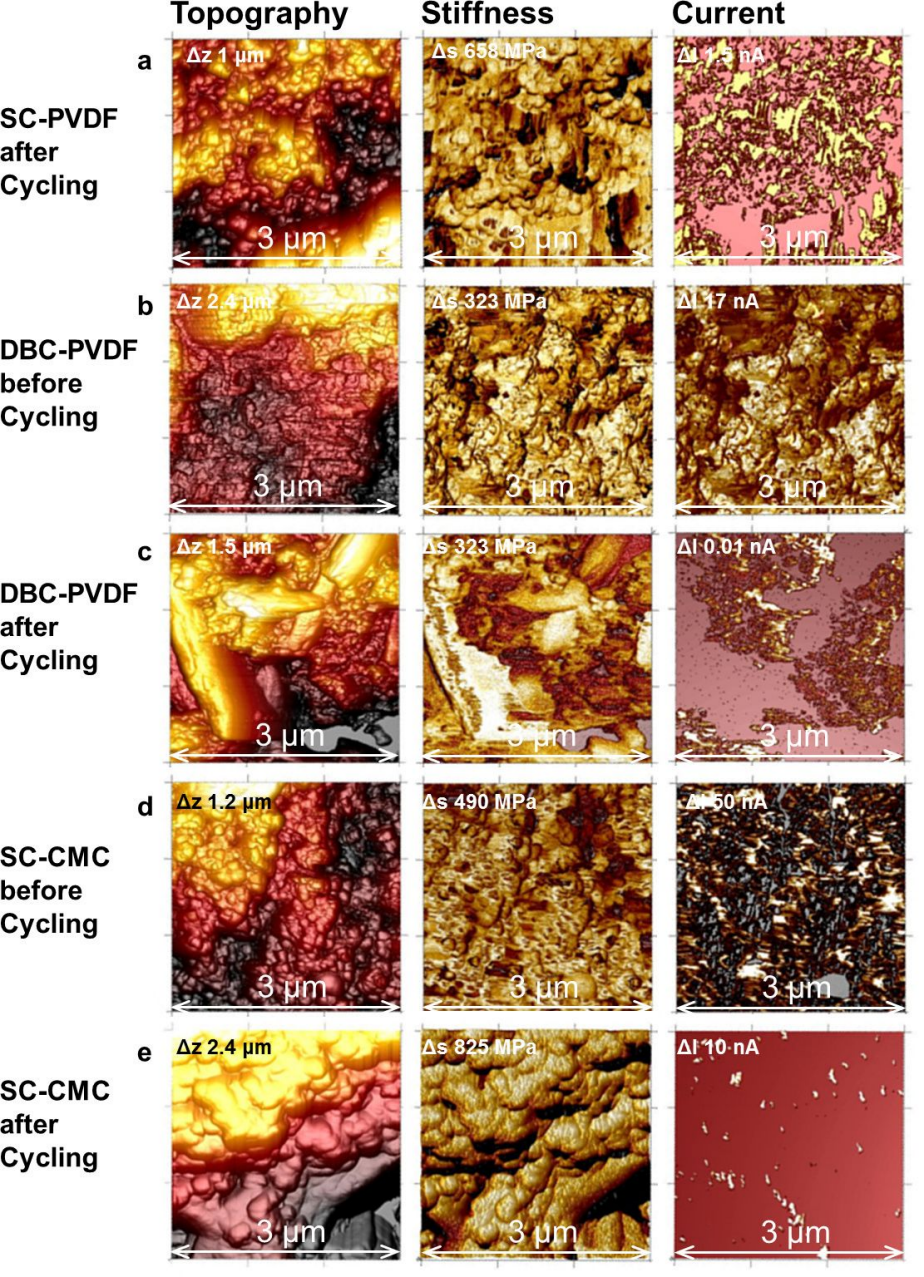
AFM image of topography, DMT modulus, and QNM™ current of a) SC-PVDF after cycling b) DBC-PVDF before cycling, c) DBC-PVDF after cycling, d) SC-CMC before cycling and e) SC-CMC after cycling. The scale bar is given as a value in the image.

After cycling the SC-PVDF sample, the grains are more defined and larger than before cycling (Figure 7a). Although, in this image the roughness is larger (Δ*z* = 1.5 µm) than before cycling, no general difference in roughness before and after cycling could be detected for this sample (see Figure 9 below). A coarsening of the finer surface structures is also visible in the SEM images (Figure 2d). A few isolated grains exhibit a high stiffness. This harder surface area is also not conductive. In general, most of the conductive regions exhibit high energy dissipation (not shown) and are quite ductile. The magnitude of the current (QNM™ current) decreased from approximately 30 nA before cycling to less than 1.5 nA (average values). In the adhesion image (not shown), three different magnitudes are distinguishable: a very high adhesion, a medium value for most of the surface and a few grains with a very small adhesion. Areas of high adhesion were non-conductive and high stiffness indicates the presence of sulfur-rich grains at the surface (see Figure 5).

In Figure 7b, topography, stiffness, and QNM™ current of the DBC-PVDF sample that was coated by the doctor blade technique are displayed. Before cycling, the height differences are larger than in the SC-PVDF sample. However, the highest current of 1.7 nA is much smaller than the 30 nA measured at the SC-PVDF sample. Most of the surface has similar hardness and low energy dissipation values. Higher currents were measured at regions with higher stiffness. Because it is not averaged, the PeakForce QNM™ current is comparable to the peak current of the PeakForce-TUNA™ mode, and thereby includes transient as well as steady-state current. Conductivity measured in stiff areas is not expected due to low conductivity of sulfur. When capacitive currents are detected, this indicates a conductive or electrochemically active layer on top of a harder sulfur grain, as shown in Figure 6. Following the results of calibration with the base materials (Figure 4 and Figure 5) and the observation of the previous measurement of SC-PVDF, the combination of high stiffness and conductivity indicates the presence of carbon-coated sulfur at the surface. The surface areas with a high energy dissipation and a low current exhibit mostly high adhesion. In the adhesion image (not shown), several small grains of approximately 50 nm size and with a very low adhesive force are visible, which appear to be very soft. These grains may consist of PVDF binder which should be soft, non-conductive, and exhibit small adhesion.

After cycling (Figure 7c), some large crystalline-like grains with lengths of approximately 2–3 μm are visible, which possess a high hardness, no conductivity, and show low deformation and energy dissipation. The region in between these crystallites exhibits a finite but very small conductivity, one order of magnitude smaller than before cycling. Here, several small conductive soft grains with low adhesion, high deformation and high energy dissipation are visible, which could be due to the presence of PVDF binder particles.

The SC-CMC sample (Figure 7d) was prepared with a different binder (carboxymethyl cellulose) and consisted of grains with approximately 100 nm size arranged in several terrace-like layers. The softer particles are quite homogeneously distributed and most of the surface is stiffer. The more ductile grains are conductive, have small adhesion, high energy dissipation and high deformation. Due to their properties, this material is most likely carbon or carbon-rich. One larger non-conductive grain at the bottom right is much harder than the surrounding regions and had high adhesion. Therefore, it is most likely a sulfur-rich particle.

After cycling (Figure 7e) the grains are enlarged, the surface is much smoother, finer particles are lost, and flat terraces are visible in the topography image. However, the total height difference has not changed significantly. The steep (almost vertical) borders of these terraces can hardly be measured by AFM and are visible as black regions. These steep and smooth features most likely represent large carbon particles with even layers visible at the borders. The surface layer stiffness is quite homogeneous. The stiffness scale of the displayed area is larger after cycling although on average the stiffness has decreased. The current decreases by more than one order of magnitude after cycling and only very small spots are still conductive. These conductive spots have higher energy dissipation and adhesion and mark some of the terrace borders.

**Figure 8:**
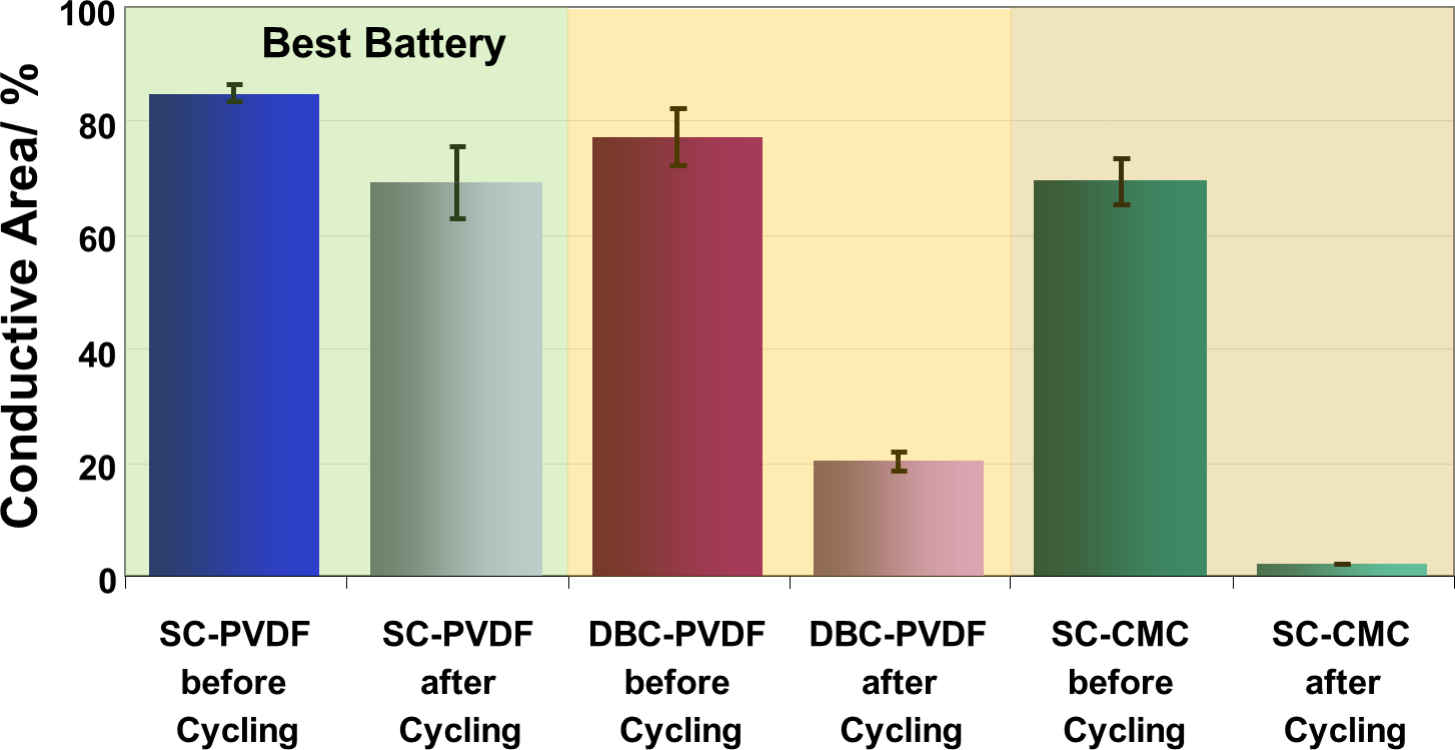
Conductive area during image acquisition under identical conditions at the cathodes before and after 50 cycles; area 3 µm × 3 µm, *U* = 0.5 V with same AFM tip.

A statistical evaluation of the mean conductive area before and after battery cycling is displayed in Figure 8. For all samples the total conductive area was always reduced upon cycling. A similar reduction of the conductive area was also reported from in situ conductive AFM measurements by Auerbach et al. [11]. This is proof for composition changes, which take place upon cycling. The smallest reduction of the conductive area was found for the SC-PVDF sample, which was in accordance with the best battery cycling performance. Much larger relative reduction is visible for DBC-PVDF and the SC-CMC samples, and for the latter an almost complete loss of the conductive area was measured. The improvement of the cycle life in SC-PVDF samples may therefore be attributed to the good electrical path and structural stability given by a well-distributed sulfur-carbon composite network.

**Figure 9:**
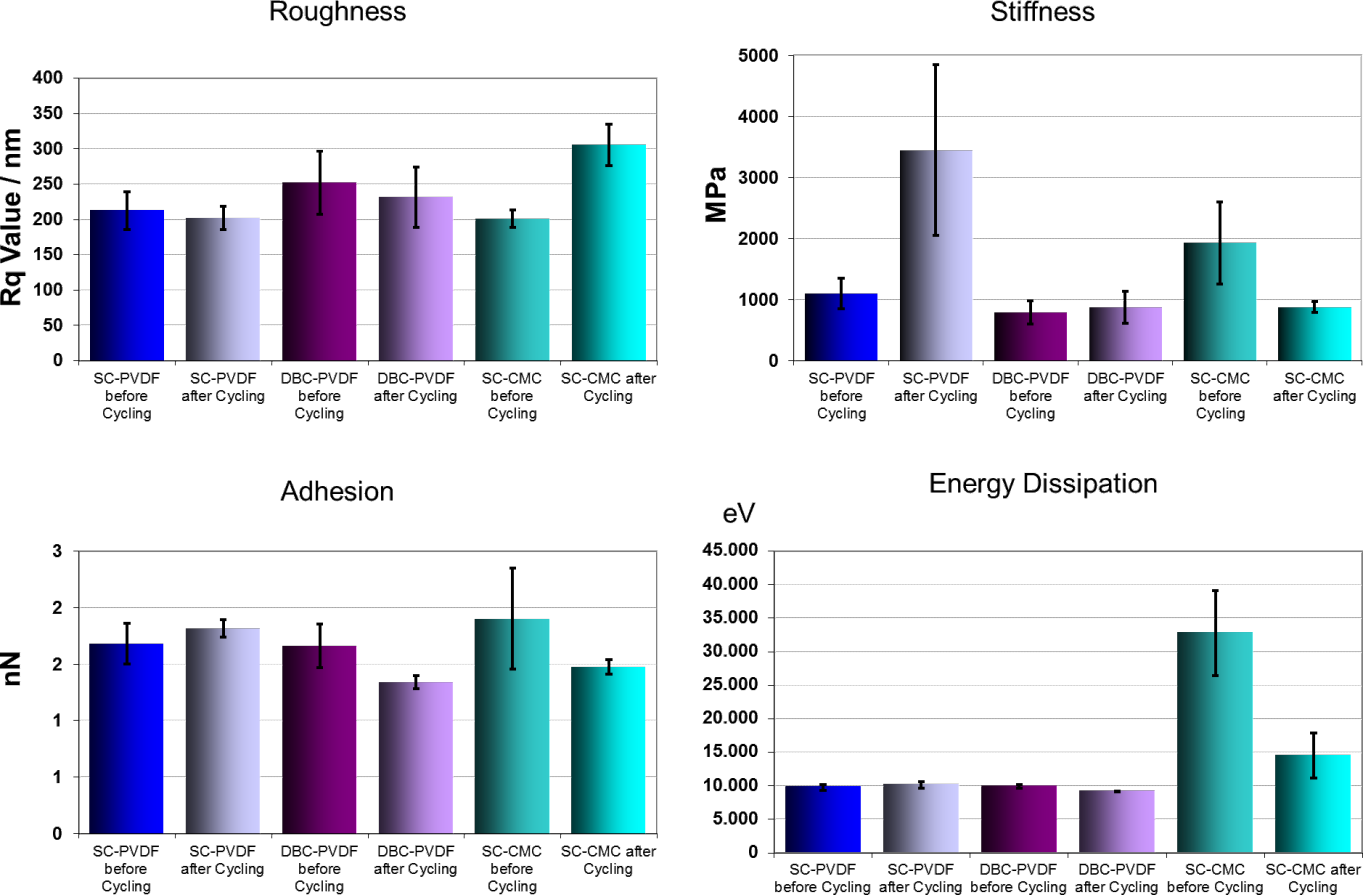
Comparison of mean values of roughness, adhesion, energy dissipation, and stiffness, from statistical evaluation of AFM images measured under identical conditions at the cathodes before and after 50 cycles; the imaged area is 3 μm × 3 μm with the same AFM tip.

In Figure 9 a comparison of the mean stiffness and the roughness of the different cathodes before and after cycling is presented. Energy dissipation indicates a non-elastic deformation of the material and is larger in the SC-CMC cathode prepared with the carboxymethyl cellulose binder compared to the cathodes containing polyvinylidene fluoride binder. The energy dissipation of the samples containing PVDF binder did not change at all. The samples with CMC binder have significantly different properties. They are much more ductile from the beginning and even after a decrease in cycling, energy dissipation is still higher for the PVDF containing samples. From the analysis of compounds it can be concluded that carbon leads to the highest energy dissipation values. As a consequence, high values indicate an initial carbon-rich surface and the reduction of energy dissipation values after cycling indicates a loss of carbon after cycling. The adhesion force is quite sensitive to the properties of a thin surface layer. A difference in surface hydrophobicity is visible in the adhesion force, which has been shown previously with other fluorine-containing materials and is expressed here in the low adhesion force of pure PVDF. Here, only small adhesion force changes are visible. A small increase in adhesion of SC-PVDF samples may point to an enrichment of PVDF after cycling but is not significantly large [35].

The differences in stiffness in the basic materials are most significant for discerning the components. The mean stiffness of the surface increased largely for the SC-PVDF sample and did not change upon cycling for the doctor-bladed PVDF-containing sample. An increased stiffness indicates an enrichment of the sulfur-containing material at the surface, which has very high stiffness values compared to pure material. In contrast, for the SC-CMC cathode with the carboxymethyl cellulose binder, the surface stiffness decreased, which indicates a fading of the initial stiff sulfur species from the surface and an enrichment of softer species. Taking into account the almost complete loss of the conductive area (Figure 8), these soft species are most likely not carbon, which would be conductive. As shown by XRD, Li_2_S_2_/Li_2_S species, which are known to form an insoluble crust at the surface, are not existent after the exposure to air. Instead, they have likely reacted to LiOH, which may still form a crust-like layer at the surface.

Although the roughness in individual images before and after cycling was different, the average roughness of the PVDF-containing samples did not change upon cycling. However, it did increase significantly in the samples prepared with CMC binder, which indicates a severe morphology change.

#### Li–S battery with optimized cathode

**Figure 10:**
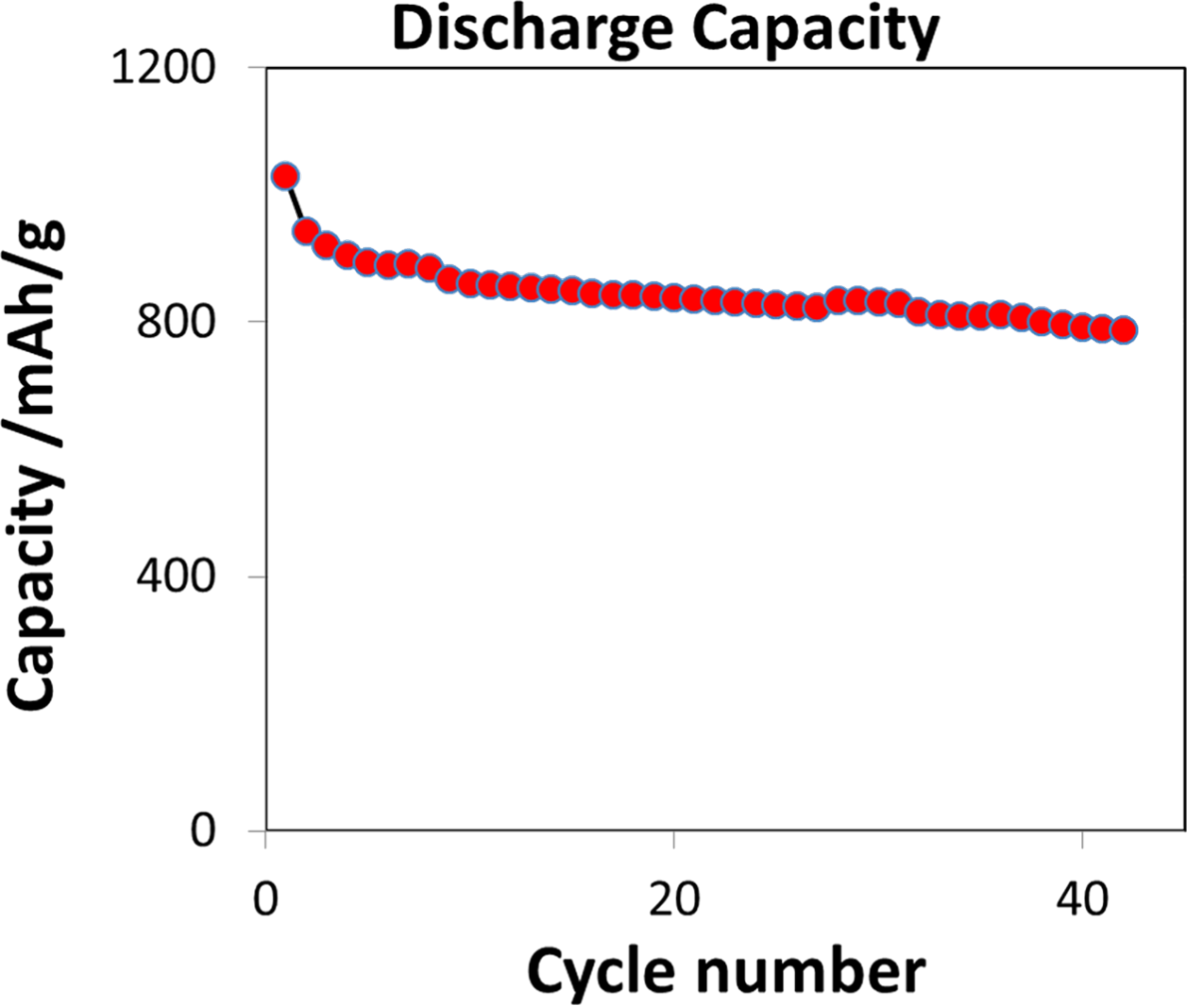
Discharge capacity of the optimized Li–S battery in the range of 2.8–1.5 V vs Li/Li^+^ at a current density of 533 mA·g(sulfur)^−1^.

From the morphology analysis the existence of large sulfur particles partly coated with a thin carbon layer was deduced. This is the reversed composition of the advantageous morphology. Therefore an additional milling step with a pearl mill to gain a finer dispersion of the superior SC-PVDF cathode material was introduced, and performance increased significantly as shown in Figure 10. The optimized battery has an initial discharge capacity of 1030 mAh·g^−1^ and after 43 cycles with a discharge rate of 533 mAh·g(sulfur)^−1^ (C/3) the capacity still measures about 800 mAh·g^−1^. SEM measurements showed finer particles and a more homogeneous surface of the samples. Further results will be reported in a forthcoming paper.

## Conclusion

In this study, it was shown that the microstructural aspects of the cathodes strongly impact the battery cycle life and the performance of the battery. SEM and AFM results confirmed a morphological change upon battery cycling, which was dependent mostly on the binder (PVDF or CMC). The superior sprayed cathode containing PVDF binder was observed to have a porous and homogeneous carbon–sulfur network structure that was the most stable upon cycling. The preparation and the nature of the binder had a significant influence on the degradation of the cathodes. Improvement of the cycle life of the sprayed cathode that contained PVDF binder could be attributed to the good electrical path and structural stability given by the well-distributed sulfur-carbon composite network. In a first approach a reversible capacity value of approximately 330 mAh·g^−1^ was retained for up to 100 cycles. After 50 cycles batteries prepared with PVDF binder showed a much better performance in accordance with a small change of the conductive area. Preparation by spray coating resulted in a significantly smaller loss of the conductive area in accordance with better battery cycling performance. Therefore, suspension spraying was proven to have the potential to be used as a high performance cathode production technique suitable for mass production.

XRD measurements showed that upon the reaction of insulating Li_2_S to LiOH, an ex-situ conductivity analysis allows the retrieval of meaningful results on the conductive surface. The ex-situ analysis is much easier in comparison to previously reported AFM measurements in a glove-box [11]. It is also very difficult to exclude small levels of water vapour, even for scrupulously performed experiments, which can endanger the reproducibility and repeatability of tests. Generally, for all samples the conductivity changed significantly upon 50 cycles as measured by AFM. Differences in transient current and steady-state current indicate the existence of disadvantageously large sulfur particles with thin carbon layers on top. An incomplete wetting of sulfur particles by carbon was also observed by AFM. Another observation was that the change of the conductive area correlated with the loss in battery performance upon cycling. The reduction of the conductive area was found to be largest for the CMC sample, only a percentage of 2.9% of the initial value was left on average, compared to 81% for the SC-PVDF sample. No correlation was found with the magnitude of current values measured by AFM. High current values correspond to highly conductive, most likely carbon-rich, areas that are not sufficient for a good battery. The surface structure changed differently for the studied samples, e.g., the sample with PVDF lost only the finer structures upon cycling, whereas for the inferior sample with CMC the overall roughness also increased. In this case, larger grains were visible and flat terraces appeared. The surface composition also changed differently upon cycling; harder grains with no conductivity were frequently observed to be present in PVDF-containing samples, and these were most likely sulfur-rich particles.

The inferior CMC-containing samples developed a carbon-rich surface after cycling. The initial stiff sulfur species were likely lost at the surface and replaced with non-conductive species, the most likely of which was LiOH, which had formed from the reaction of Li_2_S/Li_2_S_2_ in humid air.

Based on the principle advantage of the SC-PVDF preparation technique with an advantageous stability of the electronic network but too large sulfur particles, an additional milling step to get a finer dispersion was introduced in the preparation. First experiments exhibited a significant increase of the battery performance with a remaining discharge capacity of 800 mA·g(sulfur)^−1^ after 43 cycles.

These results from battery performance confirm the importance of a firm and lasting sulfur–carbon network, which depends on the nature of the binder, and the need for optimal dispersion of particles and the importance of adequate material dispersion. We could also show that advanced material-sensitive and conductive AFM techniques with their potential to analyse microstructural changes, particle size, and even chemical nature are useful to optimize battery materials.

## Experimental

### Cathode preparation

Suspensions were prepared by mixing sulfur (100 mesh, 99.5%, Alfa Aesar), carbon black Super P conductive (99%, Alfa Aesar), polyvinylidene fluoride (PVDF) binder (Aldrich) with dimethyl sulfoxide (DMSO) (≥99.9%, Aldrich) and ethanol (99.5%, VWR) 50/50 vol %. After the suspensions were thoroughly mixed for 24 h, they were sprayed on aluminium current collectors (99.45%, Alfa Aesar, thickness: 0.025 mm) by using an air-atomising internal mixing nozzle (LECHLER GmbH) piloted by a 3D axis robot. The coating was performed by spraying four layers to minimise the roughness. The thicknesses of the spray-coated cathodes were approximately 30 μm. For comparison, the cathodes were also prepared by doctor-blade coating. The spray- and hand-coated cathodes (5 cm × 5 cm) were dried under vacuum at 50 °C for 24 h. The resulting cathodes consisted of 50 wt % sulfur, 40 wt % carbon black and 10 wt % PVDF. In a similar way, cathodes containing 10 wt % carboxymethyl cellulose binder (CMC, Aldrich) were also prepared. This time, the suspension contained a 50/50 vol % ethanol/water mixture to be able to dissolve the CMC binder. Circular discs of 10 mm diameter were cut from the cathode films. The stainless-steel Swagelok® type lithium–sulfur batteries were assembled in an Ar-filled glove box by stacking together 10 mm diameter discs of lithium metal foil (99.9%, Alfa Aesar, thickness: 1.5 mm), 12 mm diameter discs of polyethylene separator (Entek, thickness: 20 μm), and the prepared cathodes. 14 μL of 1 mol/L lithium hexafluorophosphate (LiPF_6_) (99.99%, Aldrich) in tetra(ethylene glycol) dimethyl ether (TEGDME) (99.9%, Aldrich) was used as an electrolyte.

#### Cathode analysis

The charge/discharge performance of the lithium–sulfur batteries was investigated by using a Zahner IM6 electrochemical workstation with a battery cycling software. The batteries were cycled galvanostatically between 1.5 V and 2.8 V vs Li/Li^+^ at a current density of 533 mA·g^−1^ at room temperature. The morphology of the samples was investigated by SEM with a LEO Gemini (Zeiss) and AFM with a Multimode 8 Bruker system.

#### XRD measurement of Li_2_S in air

X-ray diffractograms (Figure 3) were measured in reflection mode with an X-ray diffractometer (D8 Discover GADDS) equipped with a VÅNTEC-2000 areal detector. Exposures were made with an accelerating voltage of 45 kV and a tube current of 0.650 mA. The total exposure time was 12 min [24].

#### AFM analysis

**Figure 11:**
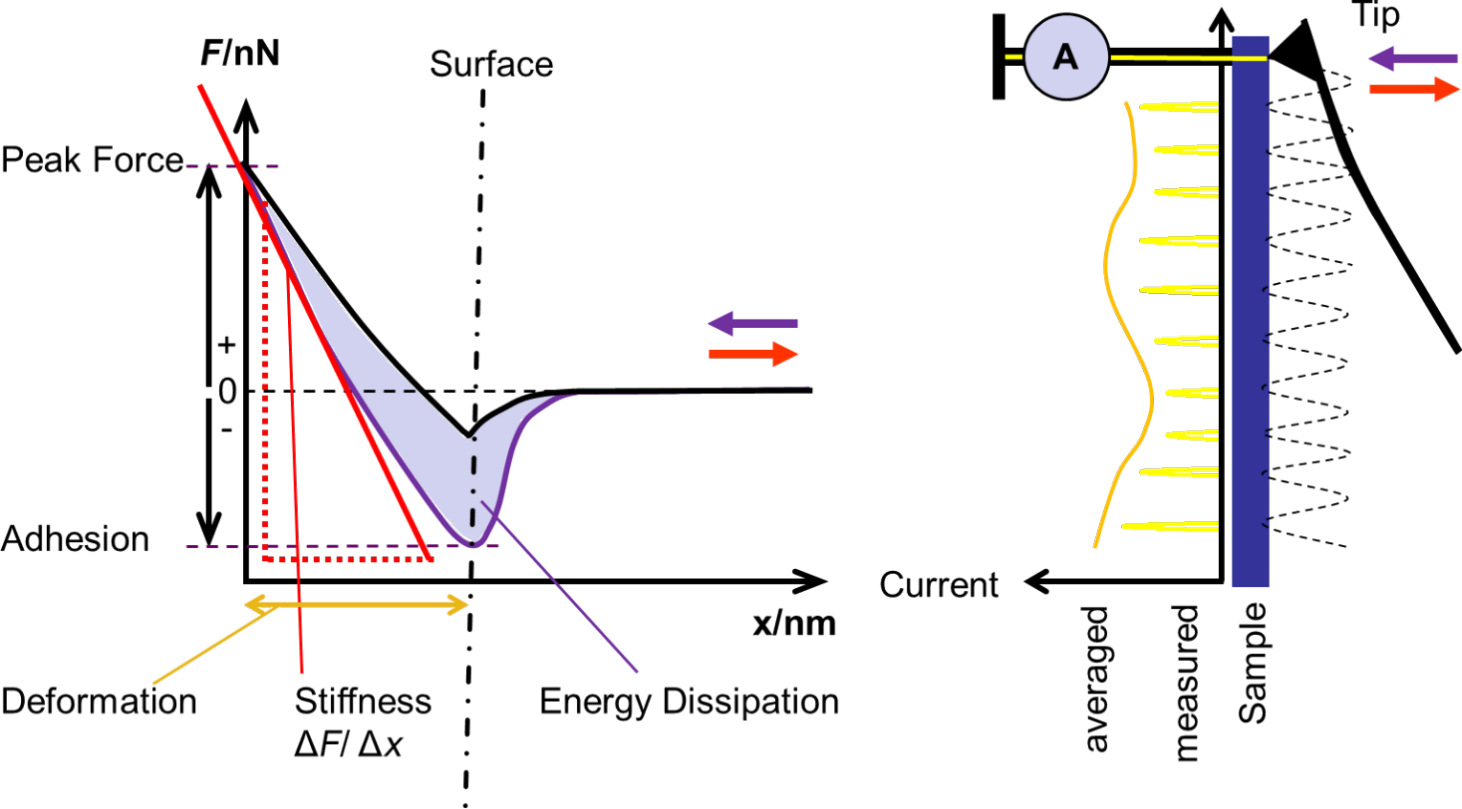
Force separation curve with scheme of evaluation of mechanical properties by AFM (left) and scheme of current measurement with time averaged signal (right) [36].

For the AFM measurements, cathodes were demounted in a reduced state and fixed with conductive silver paste onto a conductive sample holder. A Multimode 8 (Bruker Corp.) was used that is equipped with a special tapping mode, the “Quantitative Nanomechanical Peak Force” (QNM™) mode, where the force–separation curve is recorded at every image point and the topography mechanical properties (e.g., adhesion force, energy dissipation, deformation, DMT modulus or stiffness, peak force, phase shift) are simultaneously evaluated. In case of a conductive tip, the current during the contact of the tip with the sample can be recorded. A scheme of a force–distance curve is given in Figure 11 [21].

In each measurement the mapping of the pre-chosen mechanical properties is recorded. The current measurement needs to be performed with a conductive tip. In tapping mode the current flow is not continuous and a lock-in amplifier is used to derive a time averaged current (PeakForce-TUNA™ mode was used (Bruker Corp.). Two different signals were used as current output, the first of which is the averaged steady state current, named the PeakForce-TUNA™-current. The second current output is the peak current, which is recorded with a short acquisition time during the highest contact pressure and delivers higher current values due the inclusion of transient currents (acquisition time on the order of 0.001 s) [35,37]. By comparing these two current signals, transient currents can be distinguished from steady state current flow. Because the PeakForce-QNM™ current (without the use of a lock-in-amplifier) is not averaged, these results also include transient (capacitive) currents and are comparable to the peak current signal in PeakForce-TUNA™ mode. Previously, we measured conductivity in contact mode, which was associated with a significant pressure on the sample surface with the risk of surface deformation or even damage [31–32,38]. The samples were not exposed to electrolyte before cycling. The samples were investigated without an additional cleaning after cycling to preserve the surface. We also investigated the influence of contact to the electrolyte, but could reproduce the initial conductivity values afterwards. Statistical evaluation of the properties of each image was performed, yielding a histogram of the occurrence of the pixel values for a specific property. The peak occurrence value from a histogram was taken as the mean value of this property in one image. To determine the conductivity, the total conductive area in one image was evaluated. A mean value including error bars was calculated from all images measured with the same size. From five images of the same size from two different spots, at least 1 mm apart, a general average value was calculated, including the corresponding error for one standard deviation. All images used for evaluation of sample properties were recorded with an equal image size (3 µm length) and an identical AFM tip (PPP-NCHPt, NanoAndMore GmbH, spring constant: 30–50 N/m). Images measured in PeakForce-TUNA™ mode were not included in the statistical evaluations. The stiffness values of sulfur base-materials were out of the range recommended for analysis with the tip. Nevertheless, a measurement was possible and delivered a value, although a higher uncertainty can be assumed. For a differentiation of sulfur from the other components it was sufficient.
